# Relative Thrombus Burden Ratio Reveals Overproportioned Intraluminal Thrombus Growth—Potential Implications for Abdominal Aortic Aneurysm

**DOI:** 10.3390/jcm13040962

**Published:** 2024-02-08

**Authors:** Joscha Mulorz, Agnesa Mazrekaj, Justus Sehl, Amir Arnautovic, Waseem Garabet, Kim-Jürgen Krott, Hubert Schelzig, Margitta Elvers, Markus Udo Wagenhäuser

**Affiliations:** Clinic for Vascular and Endovascular Surgery, Medical Faculty and University Hospital Düsseldorf, 40225 Düsseldorf, Germany

**Keywords:** aortic aneurysm, intraluminal thrombus, aneurysm research, aneurysm growth

## Abstract

**Background:** An intraluminal, non-occlusive thrombus (ILT) is a common feature in an abdominal aortic aneurysm (AAA). This study investigated the relative progression of ILT vs. AAA volume using a novel parameter, the so-called thrombus burden ratio (TBR), in non-treated AAAs. Parameters potentially associated with TBR progression were analyzed and TBR progression in large vs. small and fast- vs. slow-growing AAAs was assessed. **Methods:** This retrospective, single-center study analyzed sequential contrast-enhanced computed tomography angiography (CTA) scans between 2009 and 2018 from patients with an AAA before surgical treatment. Patients’ medical data and CTA scans were analyzed at two given time points. The TBR was calculated as a ratio of ILT and AAA volume, and relative TBR progression was calculated by normalization for time between sequential CTA scans. Spearman’s correlation was applied to identify morphologic parameters correlating with TBR progression, and multivariate linear regression analysis was used to evaluate the association of clinical and morphological parameters with TBR progression. **Results:** A total of 35 patients were included. The mean time between CT scans was 16 ± 15.9 months. AAA volume progression was 12 ± 3% and ILT volume progression was 36 ± 13%, resulting in a TBR progression of 11 ± 4%, suggesting overproportioned ILT growth. TBR progression was 0.8 ± 0.8% per month. Spearman’s correlation verified ILT growth as the most relevant parameter contributing to TBR progression (R = 0.51). Relative TBR progression did not differ significantly in large vs. small and fast- vs. slow-growing AAAs. In the multivariate regression analysis, none of the studied factors were associated with TBR progression. **Conclusion:** TBR increases during AAA development, indicating an overproportioned ILT vs. AAA volume growth. The TBR may serve as a useful parameter, as it incorporates the ILT volume growth relative to the AAA volume, therefore combining two important parameters that are usually reported separately. Yet, the clinical relevance in helping to identify potential corresponding risk factors and the evaluation of patients at risk needs to be further validated in a larger study cohort.

## 1. Introduction

Abdominal aortic aneurysm (AAA) constitutes a significant burden to public health with a prevalence up to 12.5% in males and 5.2% in females [1]. AAA treatment remains challenging as rupture is associated with high morbidity and mortality [2]. Importantly, there is a correlation between AAA and cardiovascular disease (CVD), e.g., stroke or myocardial infarction, with a significantly higher incidence of CVD in patients with an AAA compared to other populations [3].

An AAA is considered a chronic inflammatory and atherothrombotic disease of the aortic wall, accompanied by the formation of a non-occlusive intraluminal thrombus (ILT) in most cases [4,5,6]. Despite a high ILT prevalence, there is ongoing scientific dispute regarding its role during AAA initiation and progression. Earlier studies described the ILT as reducing peak wall stress, which may protect from aortic rupture [7,8]. In contrast, more recent findings linked the presence of an ILT to increased elastolysis, a lower density of smooth muscle cells in the media layer, and increased immuno-inflammation in the adventitia [6,9].

Currently, the clinical diagnosis and initiation of open or endovascular surgical therapy is based on the AAA diameter and/or its progression, usually without consideration of the ILT and its potential risks. There have been various attempts to untangle the relationship between AAA diameter progression and the ILT. A recent study nominated the ILT as an independent predictor of AAA growth [10]. Another meta-analysis found ILT volume to be associated with AAA rupture [11]. Importantly these studies reported the maximum aortic and ILT diameter or volume separately, and only few studies have reported ILT size relative to aneurysm size [12].

Taking a step forward, we suggest a novel parameter, the so-called *thrombus burden ratio* (TBR), which is defined as a ratio between AAA and ILT volume, which may be more relevant than the ILT volume itself. Such a ratio and its changes over time have only been poorly evaluated and have never been linked to comorbidities potentially affecting ILT growth.

However, the relative changes rather than total independent parameters could have the potential to enable better timing of therapy and disease monitoring in the future and thus incorporate the TBR into the decision-making processes of clinically active surgeons.

The present study investigated the TBR and its progression in non-treated AAAs by analyzing sequential contrast-enhanced computed tomography angiography (CTA) scans and subsequently calculating the TBR for these patients. Then, CT morphological and clinical characteristics were investigated regarding their potential correlation to TBR progression.

## 2. Methods

### 2.1. Data Collection

**Figure 1:**
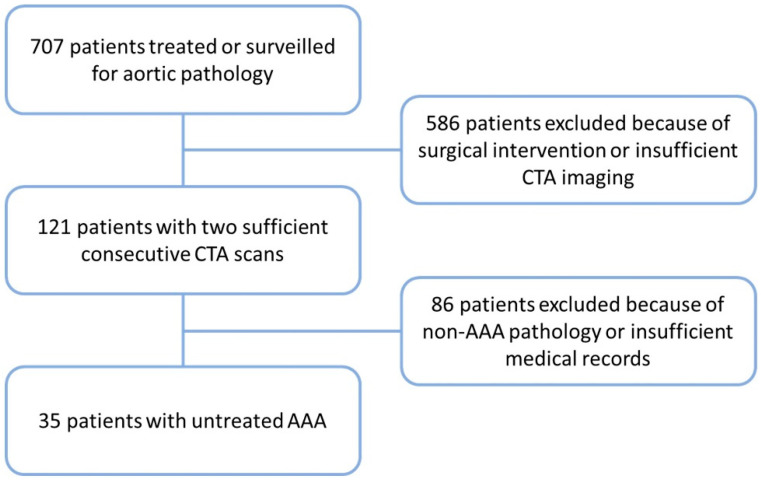
Study population. A total of 707 patients were identified with aortic pathologies between 1 January 2009 and 31 December 2018. Of those, 121 patients had two sequential enhanced computed tomography angiography (CTA) scans available. Ultimately, 35 patients were included for further measurements and analysis as study cohort based on availability of additional clinical data and sufficient CTA quality.

This retrospective study investigated 35 patients with an AAA (32 male; 3 female) treated at the Clinic for Vascular and Endovascular Surgery at the University Hospital of Düsseldorf, Germany, with two sequential CTA scans available prior to any kind of surgical treatment. Patient data were collected from 1 January 2009 to 31 December 2018 from archived medical records. Information regarding comorbidities and medication was retrieved from the patient’s medical records at the time of the first CTA scan. Out of the 707 patients treated for aortic pathologies, only 35 patients were included for ultimate analysis after applying all of the inclusion criteria (Figure 1). The study was approved by the local ethics committee at the Heinrich Heine University Düsseldorf (approval ID: 2018-2, approval date: 28 May 2018), and it followed the standards of good scientific practice and conformed to the provisions of the Declaration of Helsinki.

### 2.2. Enhanced Computed Tomography Angiography (CTA) Scan Analysis

CTA studies were acquired helically using state-of-the-art CT scanners and standard institutional protocols for CTA. Images were reconstructed at a 1–5 mm thickness. The average overall slice thickness was 2.56 ± 1.52 mm. Scans were uploaded to Osirix DICOM Viewer (Pixmeo SARL, Geneva, Switzerland) and the open-source analysis tool Horos (https://horosproject.org/) for subsequent measurements and analysis. For each patient, the following parameters were collected at both the baseline and follow-up CTA: maximum AAA diameter, total ILT and AAA volume, volume of perfused lumen, AAA wall thickness, ILT surface measured perpendicular to the centerline axis at the maximum AAA diameter, maximum ILT thickness, and AAA wall thickness at maximum ILT diameter. The region of interest (ROI) for volume measurements was defined by setting upper and lower threshold limits. Limits were defined proximally by the loss of parallelism of both aortic walls, until distally, parallelism was regained or until the aortic bifurcation occurred. AAA volume was measured with ROI-based semiautomatic segmentation.

### 2.3. Definition of TBR

ILT and AAA volume were measured using CTA scans at baseline and follow-up as mentioned above. The ratio of both measurements was defined as the TBR:

TBR = ILTvolume/AAAvolume

TBR progression was calculated as the difference between the TBR at baseline (BL) and at follow-up (FU) normalized to the baseline TBR and the time between the two sequential CTA scans:

((TBR FU − TBR BL)/TBR BL)/time period [months]

### 2.4. Spearman’s Correlation/Multivariate Logistic Regression

Spearman’s correlation was applied to analyze the relationship between morphologic parameters and TBR progression. As a next step, multivariate linear regression analysis was performed to evaluate the association of patients’ comorbidities, hemostasis-related blood parameters, and changes in AAA and ILT morphology with relative TBR progression. Here, TBR progression was set as the dependent variable, and all parameters collected were set as influencing variables.

### 2.5. Subgroup Analysis

To explore whether the TBR is different in patients with a large and/or fast-growing AAA, patients were assigned based on either their maximum AAA diameter at baseline CTA or their AAA volume increase between the sequential CTA scans. Threshold values for both were defined as follows: maximum baseline AAA diameter > 50 mm and AAA diameter progression > 1 mm^3^/month.

### 2.6. Statistical Analysis

Where applicable, testing for normality was applied as indicated in the Figure legends, as well as testing for statistical outliers. All continuous data are reported as the mean and standard error of the mean (SEM) with 95% confidence intervals (CI). Categorical data are presented as absolute frequencies with percentages. Statistical analysis was performed using SPSS Statistics Version 27 (IBM, Armonk, NY, USA) and GraphPad Software Version 9 (GraphPad Software, San Diego, CA, USA). Graphs were created with GraphPad Software Version 9 (GraphPad Software, San Diego, CA, USA).

## 3. Results

### 3.1. Patient Demographics

The study cohort consisted of 35 patients, comprising 32 males (91.4%) and 3 females (8.6%). The mean age was 68.9 ± 1.4 years (66.1–71.9) at the time of the baseline CTA scan. The time period between the two sequential CTA scans was 16 ± 15.9 months (10.4–21.3). A total of 24 (68.6%) patients had reported arterial hypertension (aHT), 9 (25.7%) patients had hyperlipidemia (HPL), 20 (57.1%) patients had a history of smoking, and 9 (25.7%) patients had a body mass index (BMI) > 30. In addition, 3 (8.6%) patients suffered from type 2 diabetes mellitus (T2D), of whom all were males. Hemostasis-related blood parameters were collected at the time of the second CTA and were within the expected norm thresholds (Table 1).

### 3.2. Morphological Parameters

Morphological parameters were analyzed on two sequential CTA scans. At baseline, the mean AAA diameter was 49.1 ± 1.7 mm (45.6–52.4), ILT volume was 67.9 ± 9.9 cm^3^ (27.9–81.0), and the AAA volume was 126.3 ±13.6 cm^3^ (98.8–153.9). Within the mean time period between the two sequential CTA scans of 16 months, there was a relative increase in AAA diameter of 12 ± 3% (4–17), in ILT volume of 36 ± 13% (11–62), and in AAA volume of 18 ± 4% (10–27), indicating an accelerated relative ILT growth when compared to AAA growth. Of interest, changes in the aortic wall thickness at the maximum ILT diameter were negligible (Table 2).

**Figure 2:**
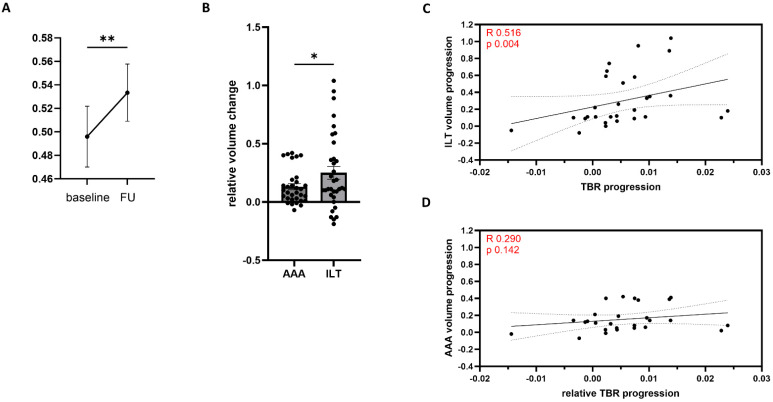
Thrombus burden ratio (TBR) and its progression. The volume of the intraluminal thrombus (ILT) and the abdominal aortic aneurysm (AAA) were measured from enhanced computed tomography angiography (CTA) scans at baseline and follow-up (FU). The ratio between ILT and AAA volume, so-called TBR, increased significantly between two sequential CTA scans (**A**). Analyzing AAA and ILT volume changes normalized to baseline (ILT/AAA volume progression) identified an overproportioned ILT volume progression suggesting ILT volume increase to be more relevant for TBR progression (**B**). Spearman’s correlation was applied to assess correlation between TBR progression and ILT (**C**) and AAA (**D**) volume progression following testing for normal distribution using Shapiro–Wilk test. R- and *p*-values are displayed in the graph. Line is simple linear regression with 95% CI indicated as dotted lines. Wilcoxon matched-pairs signed-rank test. Outliers were identified using GROUT method (Q = 5%), slightly reducing the total of *n* = 35 for some groups. Graphs are mean with SEM. * = *p* < 0.05; ** = *p* < 0.01 (**A**,**B**) or *p* indicated in graph (**C**,**D**).

Next, we investigated the TBR by calculating the ratio of ILT and AAA volume at baseline and at follow-up. Here, we observed a significant increase in TBR between the two time points (Figure 2A). The TBR increased by 11 ± 4% (1.8–20), and TBR progression was 0.8 ± 0.0.8% per month (0.8–2.4). To further evaluate whether this significant increase was due to the AAA volume or the ILT volume increase, we compared the relative changes in both parameters between the two time points. We found that the relative ILT volume change was accelerated when compared to the relative AAA volume change, suggesting an overproportioned ILT growth (Figure 2B). This finding was then confirmed by applying Spearman’s correlation. Here, ILT volume progression (R = 0.51, *p* = 0.004) correlated with TBR progression to a greater extent and this was statistically significantly when compared to AAA volume progression (R = 0.29, *p* = 0.42) (Figure 2C,D).

The Spearman’s correlation was then added for other morphologic parameters. Here, we found that the ILT thickness also correlated with TBR progression, which did not apply for AAA diameter progression (Table 3), overall, suggesting a higher relevance of the ILT for TBR progression.

### 3.3. TBR Progression in a Large and Fast-Growing AAA

**Figure 3:**
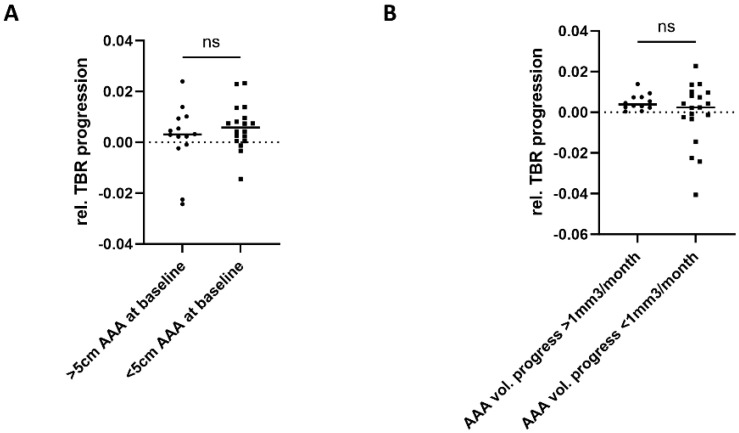
Thrombus burden ration (TBR) in large and fast-growing AAA. Patients of the study cohort were assigned either based on their maximum AAA diameter at baseline (**A**) or their AAA volume increase between the sequential CTA scans (**B**). Threshold values for both were set to AAA baseline diameter > 50 mm and AAA volume progression > 1 mm3/month. TBR progression was compared for both subgroups. There were no significant differences in TBR progression between both subgroups within the study cohort. Mann–Whitney U test was applied after identification of outliers using ROUT method (Q = 5%), slightly reducing the original total number of *n* = 35; ns: not significant.

We conducted a subgroup analysis to explore whether TBR progression was different in patients with a large or fast-growing AAA. Patients were assigned based on their baseline AAA maximum diameter or their volume increase between baseline and follow-up. We did not observe differences in TBR progression for a large vs. small AAA or fast- vs. slow-growing AAA (Figure 3).

### 3.4. TBR Progression and Potential Correlating Factors

As a next step, we performed a multivariate linear regression analysis to determine whether age, gender, cardiovascular risk factors/comorbidities, hemostasis-related blood parameters, or changes over time in morphologic AAA or ILT parameters were associated with relative TBR progression (Table 4). Among the studied characteristics, we could not identify a statistically significant association. Yet, of all the cardiovascular risk factors, T2D showed the highest regression beta coefficients (unstandardized B 0.122; standardized B 0.735). For the AAA-related morphologic parameters, AAA diameter change over time showed the highest chance of predicting TBR progression (unstandardized B 0.594; standardized B 2.356), though this was not statistically significant.

## 4. Discussion

The role of ILT in AAA has gained significant interest in recent years. Yet, we are still at the beginning of unveiling its detailed role in the disease. This retrospective study focused on the relationship between ILT and AAA volume progression over time, and the ratio between those two parameters was defined as the thrombus burden ratio (TBR). We found that the growth of an ILT contributes to this ratio to a greater extend when compared to changes in AAA volume, indicating a faster ILT growth over time than overall AAA growth. However, there was no difference in the TBR progression with a fast- vs. slow-growing AAA or an initially large- vs. small-sized AAA. Also, due to the small sample size, we were not able to identify clinical or morphological parameters directly associated with TBR changes.

While the correlation between the risk of AAA rupture and the maximum AAA diameter is well established [13], this linear relationship does not seem to apply for the ILT. For example, recent studies have suggested a thin ILT as an individual risk factor for increased AAA growth and risk of rupture with potential implications for the timing of surgical treatment [14,15]. While this suggests a potentially protective role of the ILT, other studies have taken a fundamentally different view of the role of the ILT in an AAA.

Following such a theory, it is implied that as soon as an undefined threshold of thickness is reached, the potentially protective effects of an ILT are at least equalized by its microstructural composition and the associated proteolytic effects. The sum of these proteolytic effects results in a weakening of the AAA wall, which in turn, may contribute to accelerated AAA growth [4]. Interestingly, this mechanistic role of the ILT is supported by the observation that a high ILT burden was linked to decreased aortic wall strength and an elevated risk of a small AAA rupture [16]. This has also been linked to a reduced supply of oxygen to the aneurysmal wall due to the large ILT mass [17]. This is further supported by findings indicating a ruptured AAA to have a larger diameter and larger ILT volume when compared to an intact AAA [18]. Pointing in the same direction, reports indicate that the presence of an ILT is associated with an increased risk of rupture and faster ILT growth, especially in a small-sized AAA of ≤50 mm [19]. In contrast to these observations, the data from this study show disproportionate ILT vs. AAA growth (TBR), although there was no difference in small AAAs (<50 mm) vs. large AAAs (>50 mm).

Of note, most studies available in the literature to date have one important limitation in common. In most cases, ILT and AAA volume and/or diameter are considered separately from each other; a relative relationship between these two parameters is rarely established, although there is a conclusive argument for that. Here, the implementation of the TBR may be useful for future studies, since this parameter describes the relative ILT growth in relation to the AAA growth over a period of time and could therefore be a decisive future predictor for the determination of an individual’s rupture risk or AAA growth rate as well as for the quality of care in regard to the complication rate after open or endovascular therapy [20]. This seems particularly likely, since a large ILT deposition was shown to negatively affect long-term results following endovascular therapy [21].

The results of this study could not establish a direct correlation between any clinical or morphological parameters and the TBR, likely due to the limited sample size. A previous study based on Danish registry data also investigated the role the ILT in an untreated AAA based on ultrasound-derived data. Here, the authors found a small, yet statistically significant correlation between the relative ILT maximum area and AAA growth in their multivariate linear regression model, which we were not able to identify. However, this was carried out in a vastly larger cohort study of 400 patients [22]. In the present study, we were able to gather CTA follow-up data of untreated AAAs, which to our knowledge, has never been published to this extent; the cohort size in this monocentric study hardly allows for any strong conclusions to be drawn regarding the identification of factors affecting ILT growth.

Identification of such factors may be tough in other regards as well. As mentioned above, it is unclear if a large ILT is protective or harmful and whether there is a potentially beneficial threshold for AAA development, which further complicates the process of identifying clear associations.

Conclusively, the multifactorial influence of several parameters in their interaction appears to be the most likely contributor to the TBR. However, some parameters may appear more important than others. For example, T2D and the red blood cell (RBC) count showed the most robust correlation with the TBR. Such a correlation seems particularly plausible against the background of a known increased platelet activation in T2D. In this context, hyperglycemia may have direct osmotic effects by activating platelet GP IIb/IIIa and *p*-selectin, with protein kinase C (PKC) activation contributing to platelet activation [23,24,25]. Also, the lack of insulin in patients with diabetes may contribute to enhanced platelet activation as insulin binds to the insulin receptor (IR) located on the platelet surface and activates insulin receptor substrate 1 (IRS-1) via tyrosine phosphorylation. This, in turn, mediates its association with Gi protein α (Giα)-subunit [25,26,27], which ultimately increases intraplatelet cyclic adenosine monophosphate (cAMP), resulting in decreased platelet activity [28].

The roles of RBCs in hemostasis and thrombosis have become increasingly clear in the recent years. Multiple mechanistic studies suggest that RBCs can actively promote thrombus formation and should no longer be considered as a passive bystander [29]. To this end, multiple hemorheological effects, effects on platelet reactivity, and interactions with the vessel wall are described, although their relevance for ILT formation in AAA has not been investigated at this moment in time [30]. Based on the data presented in this study, the authors strongly encourage further mechanistic studies to identify possible relevant pathways that may contribute to the TBR and that are also beneficially targetable.

If an attempt is now made to finally shed more light on the possible clinical relevance of the TBR, the authors consider two scientific questions to be pressing. On the one hand, the extent to which the TBR may serve as a possible predictor of AAA rupture or increased disease-associated complications needs to be evaluated. Here, a prospective, multicentric study design is needed for patients undergoing AAA surveillance on a larger scale, as introduced in this preliminary study involving multiple institutions. Also, patients with an incidental AAA diagnosis during CT scans for other medical reasons should be included and prospectively followed up. On the other hand, it needs to be verified whether the TBR can predict outcomes after AAA treatment, both endovascular and open. In this context, the TBR may prove useful, as there is accumulating evidence that the ILT affects outcomes after EVAR and TEVAR [21,31]. Again, larger-scaled studies are needed to explore this potential.

This study has various limitations. First, the stringent inclusion criteria have limited the cohort size to 35 patients, since follow-up CT is not regularly available in patients with a large AAA. The small sample size needs to be considered when drawing conclusions from the study. Also, the single-center study design and the retrospective setting may have further biased our findings.

## 5. Conclusions

In summary, we found that the TBR increases during AAA development indicating an overproportioned ILT vs. AAA volume growth with no preference with regard to a small vs. large and a fast- vs. slow-growing AAA. Nevertheless, the TBR may serve as a useful parameter in future studies since it incorporates several factors in one parameter. Factors that contribute to increased ILT growth or the TBR are not yet known but appear mechanistically interesting.

## Figures and Tables

**Table 1:** Patient characteristics. Data are shown as mean ± standard error of the mean (SEM) with 95% confidence interval (95% CI) or absolute frequency with percentages (%) (*n* = 35).

Parameter	Absolute Frequencies (%) Mean ± SEM
Demographics and Comorbidities	*n* = 35 total
Male gender	69.4 ± 1.4
Age	32 (91.4)
aHT	3 (8.6)
HLP	24 (68.6)
DMTII	9 (25.7)
BMI > 30	20 (57.1)
History of smoking	9 (25.7)
Blood parameters at second CTA	
HCT	39.44 ± 1.31
Hb	13.10 ± 0.44
RBC	4.36 ± 0.18
PLC	219.60 ± 12.89
WBC	10.09 ± 1.00
Quick	90.71 ± 2.92
INR	1.10 ± 0.03

DMTII: type II diabetes mellitus, aHT: arterial hypertension, HLP: hyperlipoproteinemia, BMI: body mass index, HCT: hematocrit, Hb: hemoglobin, RBC: red blood cell count, PLC: platelet count, WBC: white blood cell count, INR: international normalized ratio.

**Table 2:** Morphological parameters on enhanced computed tomography angiography (CTA)-based analysis. Various image-based morphologic parameters for abdominal aortic aneurysm (AAA) and intraluminal thrombus (ILT) were measured from two sequential CTA scans at baseline and at follow-up. Data are shown as mean ± standard error of the mean (SEM) and 95% confidence interval (95% CI). Relative changes between the two time points are reported by normalizing to baseline (*n* = 35).

Parameter	All (*n* = 35)		Relative Change vs. Baseline
	Baseline CTA Scan	Follow-Up CTA Scan	
ILT volume (cm^3^)	67.9 ± 9.9 (27.9–81.0)	80.6 ± 9.4 (61.6–99.6)	0.36 ± 0.13 (0.11–0.62)
AAA volume (cm^3^)	126.3 ± 13.6 (98.8–153.9)	145.3 ± 14.1 (116.6–173.9)	0.18 ± 0.04 (0.10–0.27)
Thrombus burden ratio (TBR)	0.5 ± 0.03 (0.44–0.55)	0.53 ± 0–02 (0.48–0.58)	0.11 ± 0.04 (0.02–0.20)
AAA diameter (mm)	49.1 ± 1.7 (45.6–52.4)	53.5 ± 1.6 (50.2–56.8)	0.12 ± 0.03 (0.04–0.17)
ILT surface at max. aortic diameter (cm^2^)	9.6 ± 1.0 (7.5–11.6)	11.7 ± 1.0 (9.6–13.8)	0.51 ± 0.22 (0.05–0.96)
ILT thickness (mm)	17.2 ± 1.5 (14.1–20.3)	21.3 ± 1.7 (17.9–24.7)	0.55 ± 0.28 (−0.01–0.11)
Aortic wall thickness at max. ILT diameter (mm)	1.9 ± 0.06 (1.7–2.0)	1.9 ± 0.05 (1.8–2.0)	0.05 ± 0.03 (−0.01–0.01)

ILT: intraluminal thrombus, AAA: abdominal aortic aneurysm, max.: maximum.

**Table 3:** Spearman’s correlation. Spearman’s correlation was applied to analyze various morphological parameters and their relative progression on two sequential enhanced computed tomography angiography (CTA) scans on whether they are associated with the thrombus burden ratio (TBR) progression. R- and *p*-values are displayed together with the 95% confidence interval (95% CI). Calculations were performed after identifying statistical outliers using the ROUT method (Q = 5%), reducing the overall numbers from *n* = 35 for some parameters.

Parameter	R	95% CI	*p*-Value
ILT volume change	0.51	V0.17–0.74	0.004
AAA volume change	0.29	−0.11–0.61	0.142
AAA diameter change	0.11	−0.28–0.47	0.577
ILT surface at max. aortic diameter change	0.31	−0.09–0.63	0.114
Aortic wall thickness at max. ILT diameter change (mm)	−0.15	−0.50–0.23	0.410
ILT thickness change	0.44	0.05–0.71	0.024

ILT: intraluminal thrombus, AAA: abdominal aortic aneurysm, max.: maximum.

**Table 4:** Multivariate logistic regression analysis for comorbidities and blood parameters. Multivariate logistic regression analysis was applied to test the association of co morbidities, blood parameters at baseline, and CTA morphologic parameters with TBR progression. Data are reported with coefficient, standard error, *p*-value, and upper and lower 95% confidence intervals (95% CI) (*n* = 35).

	Unstandardized Coefficient		Standardized Coefficient				
Model	B	Standard Error	Beta	t	*p*-Value	Upper 95% CI	Lower 95% CI
Constant	−0.010	0.470		−0.021	0.984	−1.094	1.074
**Demographics and Comorbidities**							
Gender	0.045	0.091	0.271	0.498	0.632	−0.164	0.254
Age	−0.001	0.003	−0.218	−0.426	0.681	−0.008	0.005
aHT	0.007	0.044	0.072	0.163	0.874	−0.095	0.109
HLP	0.005	0.039	0.043	0.117	0.910	−0.086	0.095
T2D	0.122	0.084	0.735	1.460	0.183	−0.071	0.316
BMI > 30	0.030	0.043	0.285	0.710	0.498	−0.068	0.129
History of smoking	−0.011	0.030	−0.110	−0.353	0.733	−0.080	0.059
**Blood parameters at second CTA**							
HCT	−0.003	0.007	−0.560	−0.468	0.652	−0.020	0.014
Hb	−0.003	0.020	−0.161	−0.143	0.890	−0.050	0.044
RBC	0.032	0.020	0.721	1.612	0.146	−0.014	0.078
PLC	0.000	0.000	−0.341	−0.837	0.427	−0.001	0.000
WBC	−9.076 × 10^7^	0.005	0.000	0.000	1.000	−0.011	0.011
Quick	0.001	0.002	0.223	0.255	0.805	−0.005	0.006
INR	0.035	0.173	0.110	0.199	0.847	−0.365	0.434
**AAA and ILT morphology**							
Rel. AAA diameter	0.594	2.468	2.356	0.241	0.816	−5.097	6.285
Rel. ILT volume	−0.043	0.117	−0.685	−0.371	0.720	−0.313	0.226
Rel. AAA volume	−0.293	0.331	−1.525	−0.884	0.403	−1.057	0.471
Rel. ILT surface at max. aortic diameter	0.045	0.100	1.253	0.447	0.667	−0.186	0.275
Rel. ILT max. thickness	0.001	0.018	0.048	0.076	0.941	−0.041	0.044
Rel. Aortic wall thickness at max. ILT diameter	0.141	1.939	0.505	0.073	0.944	−4.330	4.611

aHT: arterial hypertension, HLP: hyperlipoproteinemia, T2D: type II diabetes mellitus, HCT: hematocrit, Hb: hemoglobin, RBC: red blood cell count, PLC: platelet count, WBC: white blood cell count, INR: international normalized ratio.

## Data Availability

The underlying data are available from the corresponding author upon reasonable request.
